# Developing Fine-Grained Actigraphies for Rheumatoid Arthritis Patients from a Single Accelerometer Using Machine Learning

**DOI:** 10.3390/s17092113

**Published:** 2017-09-14

**Authors:** Javier Andreu-Perez, Luis Garcia-Gancedo, Jonathan McKinnell, Anniek Van der Drift, Adam Powell, Valentin Hamy, Thomas Keller, Guang-Zhong Yang

**Affiliations:** 1 The Hamlyn Centre, Imperial College London, London SW7 2AZ, UK; javier.andreu@imperial.ac.uk; 2 School of Computer Science and Electronic Engineering, University of Essex, Colchester CO4 3SQ, UK; 3 Clinical Innovation & Digital Platforms; Projects, Clinical Platforms & Sciences, GSK, Stevenage SG1 2NY, UK; luis.x.garcia-gancedo@gsk.com (L.G.-G.); valentin.x.hamy@gsk.com (V.H.); 4 Emerging Platforms, Platform Technology & Science, GSK, Stevenage SG1 2NY, UK; jonathanmckinnell@hotmail.com (J.M.); Thomas.h.keller@gsk.com (T.K.); 5 Tessella, Altran’s World Class Center for Analytics, Stevenage SG1 3QP, UK; anniek.vanderdrift@tessella.com (A.v.d.D.); adam.powell@tessella.com (A.P.)

**Keywords:** rheumatoid arthritis, actigraphy, continuous monitoring, machine learning

## Abstract

In addition to routine clinical examination, unobtrusive and physical monitoring of Rheumatoid Arthritis (RA) patients provides an important source of information to enable understanding the impact of the disease on quality of life. Besides an increase in sedentary behaviour, pain in RA can negatively impact simple physical activities such as getting out of bed and standing up from a chair. The objective of this work is to develop a method that can generate fine-grained actigraphies to capture the impact of the disease on the daily activities of patients. A processing methodology is presented to automatically tag activity accelerometer data from a cohort of moderate-to-severe RA patients. A study of procesing methods based on machine learning and deep learning is provided. Thirty subjects, 10 RA patients and 20 healthy control subjects, were recruited in the study. A single tri-axial accelerometer was attached to the position of the fifth lumbar vertebra (L5) of each subject with a tag prediction granularity of 3 s. The proposed method is capable of handling unbalanced datasets from tagged data while accounting for long-duration activities such as sitting and lying, as well as short transitions such as sit-to-stand or lying-to-sit. The methodology also includes a novel mechanism for automatically applying a threshold to predictions by their confidence levels, in addition to a logical filter to correct for infeasible sequences of activities. Performance tests showed that the method was able to achieve around 95% accuracy and 81% F-score. The produced actigraphies can be helpful to generate objective RA disease-specific markers of patient mobility in-between clinical site visits.

## 1. Introduction

Rheumatoid arthritis (RA) is a chronic systemic disease that typically affects adults between the ages of 30 and 60 [1], leading to a life disability in which patients describe increasing pain in several joints over time. This disease has a prevalence of 0.24% that varies between 0.3% and 1% in the developed countries [2,3]. Some studies have also concluded that the risk of mortality of RA patients is approximately 38% higher than the general population, with an increased risk of 55% for women [4]. RA patients are likely to exhibit worse functional status and quality of life than those affected by osteoarthritis [5,6]. A high percentage of RA sufferers (around 50%) are unable to hold a full time job due to the considerable impact of the disease on their daily lives [3]. Research also suggests that the decrease in physical function correlates with a high prevalence of depression and anxiety [7,8]. Thus far, the effect of the disease is assessed based on sparse time-point check-outs and questionnaires during ordinary appointments.

Recent advances in wearable sensing allow continuous patient monitoring in an unobtrusive way, providing a potential source for estimating actigraphies [9,10,11]. The use of additional motion sensors such as gyroscopes, magnetometers and inertial measurement units provides a rich source of kinematic information to obtain effortless actigraphies with high precision and accuracy [12,13]. Clinical studies using several accelerometers have been proposed for studies lasting from one to three days [14]. However, due to battery, life long-term continuous clinical mobility experiments, performed during periods of several weeks up to months, have employed of single inertial sensors with on-board storage memory [15]. Consequently, in the present study, inertial data is also collected from a single tri-axial accelerometer attached to the skin in the position of the fifth lumbar vertebra (L5). The effects of RA can lead to pain in different parts of the body therefore limiting the range of movement, as well as articular sensitivity and function. Hence, certain patients may exhibit distorted mobility patterns, which can make the recognition of a determined activity from a signal segment difficult.

Actigraphies provide a closer look at some relevant factors in RA management such as alteration on patient life-styles and changes on their circadian rhythms [16,17,18,19]. However, these factors cannot be monitored accurately by ordinary appointments that are sparse in time by nature. Manual actigraphies are prone to subjectivity and ambiguity, hence the importance of automated methods for continuous assessment. Fine-grained actigraphies able to predict physical activities from accelerometer signals are key to elucidate novel digital endpoints. In this work a laboratory study is performed on rheumatoid arthritis patients to evaluate the capabilities of the recordings from a single sensor to recognize a set of human physical activities. A set of desirable activity tags are defined, namely sedentary activities (sitting, lying, and standing), acyclic activities (walking), short-timed transitions (sitting-to-standing, standing-to-sitting, lying-to-sitting, sitting-to-lying), and composite activities (lying-to-standing, standing-to-lying). In order to predict these activity tags over the acceleration data from a single sensor, a “hierarchical dichotomy” is proposed and described in detail. Several alternative machine learning methods are used and performance results are compared. Additional functions to filter for unknown activities as well as a logical filter to correct for an unfeasible sequence of transitions are also introduced.

## 2. Background and Motivation

While questionnaires and self-administered surveys of behaviours, mobility and quality of life have been largely used in clinical research in RA, their susceptibility to error and subjectivity have stimulated the utilisation of wearable sensors [20]. A physical activity assessment with more than a hundred RA patients has been attempted with an accelerometer attached to the waistline of the right hip in line with the right axila [21]. The accelerometer was used to count activity bouts occurring in a ten-minute sequence. Activity bouts can be used as a surrogate measure of the relative amount of activity; however, it does require long periods of recording and the specific activity the patient is performing is undetermined. In [22], a study with a cohort of 98 RA patients used an accelerometer sensor attached to the arm to determine an actigraphy based on the detection of four different levels of activity (sedentary, very light, light and moderate) based on a scale related to energy expenditure. The time that a patient spends performing each activity level is used as a measure of clinical interest. Although activity levels provide an insight into the amount of physical activity that patients perform during the day, a higher level of detail on the actigraphy could provide additional information on the effects that disease manifestation might have on patient behaviours, circadian rhythms and daily activity patterns.

**Figure 1:**
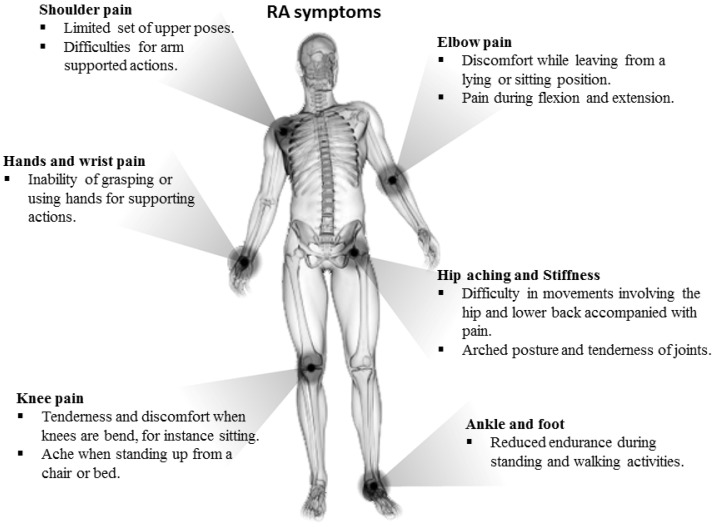
Most common mobility impairments for moderate to severe RA patients.

Generating actigraphies from RA patients is challenging as they may exhibit an amalgam of anomalous movement patterns. Issues in mobility have been described in RA patients for different activities such as those arising from a chair or bed as a result of poor gripping and trunk flexion, low range of movement and pain [23,24] or difficulty walking due to weak and painful knees [23], disturbance during sitting or lying due to stiffness and hip pain [25], and difficulty of maintaining a standing upright position [25]. As a difference to healthy subjects who might exhibit a quite similar kinetic and kinematic pattern when performing activities and transitions, disorders in activity patterns can be presented at different levels of intensity with respect to the level of the disease. Most common symptoms are displayed in Figure 1. Moreover, symptoms vary across patients and such heterogenity may accentuate the differences between patterns even further. The key challenge that this paper aims to address is to provide a method that can produce accurate fine-grained actigraphies from RA patients with moderate to severe levels of disease. In this paper, we present a method that have worked effectively in a clinical setting.

## 3. Materials and Methods

### 3.1. Experimental Settings

**Figure 2:**
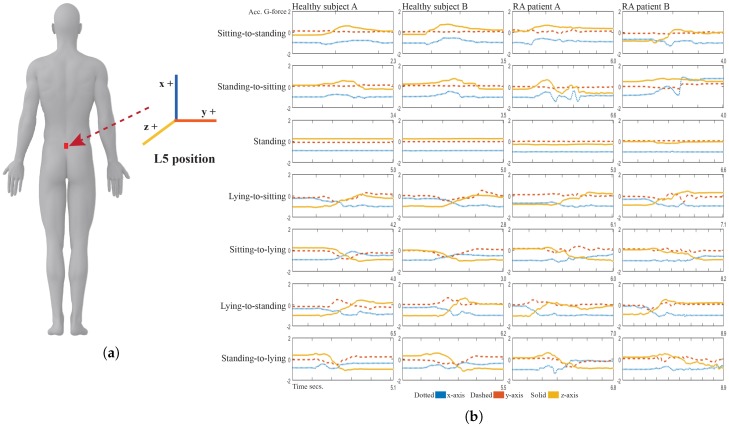
(**a**) Position of the accelerometer sensor and axes. (**b**) Signals captured by the accelerometer in the L5 position for seven exemplar activities. The signal plots depict the differences in signal pattern for healthy subjects and RA patients.

A mobility lab experiment was performed at GSK’s clinical unit in Addenbrookes Hospital (Cambridge, UK), where ten patients with moderate to severe RA and twenty healthy volunteers (HV) were asked to perform a circuit of daily living activities. This consisted of ten tasks denoted as sitting, lying, standing, walking and the transitions sit-to-stand, stand-to-sit, lie-to-sit, sit-to-lie, lie-to-stand and stand-to-lie. The tasks were performed according to a circuit that consisted of the following stations: (1) walking normally at different speeds with turning; (2) standing, sitting on different types of seats then standing; (3) lying, sitting and lying; (4) lying, standing and lying and (5) lying normally for a short period of time. Stations 2 to 4 with transitional tasks were repeated several times. The subjects were asked to perform the activities at their own pace and according to their own habit. They were conducted under normalized settings with conventional furniture such as commonly designed (unarmed) chairs, couches and beds. The sensor was attached with a clinical plaster to the fifth lumbar vertebra, as shown in Figure 2a. The mobility sensor consisted of a wearable three-axis accelerometer logger (Axivity AX3, Axivity Ltd., York, UK) [26]. The accelerometer was configured to sample data at 100 Hz.

Figure 2b shows the typical pattern of inertial signals captured from the accelerometer HV and RA patients where it can be observed that patterns on healthy volunteers are more homogeneous than the ones obtained from the RA patients. These differences in patterns might be produced as a result of a deficient mobility due to the lower range of motions as well as the patient’s trembling and balancing to avoid pain. Additionally, the patient might end up resting in an unconventional position while sitting or lying and might also exhibit a hunched behaviour while standing or walking.

### 3.2. Inertial Data Pre-Processing

For pre-processing, bias and scale parameters are computed for the calibration of the inertial sensor readings using a Gauss–Newton nonlinear optimization method based on the recordings from nine different static positions [27]. Since we only rely on one accelerometer sensor to develop the actigraphy, besides the sensed g-forces (Acc), three additional measurements of the rotation angles (i.e., roll, pitch, and yaw) are extracted, which we denote with the symbol *O*. Artifactual high-frequency noise is reduced by applying third-order median filter and the effect of the earth gravitational forces is reduced by low-pass filtering the signal up to 1 Hz and subtracting the resulting filtered signal (gravitational component) from the original inertial data, leaving just body acceleration. An estimation of the body orientation was performed by band-pass filtering the raw acceleration between 0.5 Hz and 15 Hz and then estimating the roll, pitch and yaw orientations by solving the following equations [28]:

(1) $$O_{roll}=\arctan\left(Acc_{x},\;sign\left(Acc_{z}\right)\;\sqrt{Acc_{z}+0.01\;Acc_{x}^{2}}\right)\;180/\pi \;,$$

(2) $$O_{pitch}=\arctan\left(-Acc_{x}\;,\;\sqrt{Acc_{y}^{2}+Acc_{z}^{2}}\right)\;180/\pi \;,$$

(3) $$O_{tilt}=\arctan\left(\frac{Acc_{z}}{\sqrt{Acc_{x}^{2}+Acc_{Y}^{2}+Acc_{z}^{2}}}\right)\;180/\pi \;,$$

where $sign(Acc_{z})$ has a value of +1 if $Acc_{z}$ is non-negative and −1 if $Acc_{z}$ is negative, in the second term of the roll a 0.01 fraction of the $Acc_{x}^{2}$ is added to avoid making this term zero. The orientation computed by Formulae (1)–(3) is not supported by the uses of magnetometers and gyroscopes, hence it can only be considered as an initial estimation. Nevertheless, they can still provide information in addition to the body acceleration, which is helpful for the activity classification. From the body acceleration and estimated body orientation, signal descriptors are computed over a sliding window for a period of three seconds with one-second overlap. The extracted descriptors are applied to both body acceleration and orientation for each of the three signal axes. These descriptors are: mean, variance, root mean square (RMS), number of peaks, difference in magnitude between the maximum peak and the minimum peak, number of through, average distance between peaks and troughs, zero-crossings, coefficient of variation, interquartile range, entropy, cross-correlation between the axes, power of the signal, dominant frequency, power of the signal around the dominant frequency, mean and variance of the signal magnitude area, and mean and variance of the signal magnitude vector. The vector of input sample data and the vectors of activity labels for each sample are denoted in this paper as $X=[x_{1}^{M},x_{2}^{M},x_{3}^{M},\ldots ,x_{n}^{M}]$ and $Y=[y_{1},y_{2},y_{3},\ldots ,y_{n}]$, where *n*, M⊆{1,…,m} is the subset of dimensions of the input vector x, and *m* is the total number of descriptors. For the sake of simplicity, we will refer to *x* as the input vector $x^{M}$ in the following sections.

### 3.3. Subset Selection and Subspace Mapping

The next step is to select a subset of predictors from the ones already computed from Section 3.2 and projecting them into a new subspace that maximizes the separability among the different classes of activities. As the only available information is provided from a single sensor, the data is pre-processed to discover meaningful representations that could ease the recognition. First, a classifier model from the ones described in Section 3.4 is incorporated and a feature selection approach starting from a single descriptor selected at random, appends or removes variables (i.e., descriptors) to and from the dataset that is used to train the classifier according to a predefined criterion based on recognition accuracy. Specifically, we employ a sequential forward floating selection (SFFS) [29], which yields feasible time-complexity while considering a reasonable amount of combinations. The resulting subset of predictors will be used to compute a new optimal set of features used to train a classifier. Two types of mapping learning models are adopted in this work, the first being based on metric learning mapping and the second on deep learning.

**Metric learning mapping:** This method consists of estimating the hyperparameters of a metric (distance) that collapses the neighborhood of all points from the same class to a single location in the feature space. The neighborhood of a sample is defined by a metric, more specifically a distance function between the points. This procedure is commonly known as metric learning. An optimization step of the hyperparameters for a *maximally collapsing metric* (MCM), in particular the positive singular matrix of the Mahalanobis distance, is presented in [30]. This method is aimed at minimizing the Kullback–Leibler (KL) divergence between a bi-level distribution of the class labels and a conditional distribution over the points,

(4) $$\underset{A}{min}\sum KL\left[p_{0}\left(j|i\right)|\hat{p}\left(j|i\right)\right],$$

where the distribution $p_{0}$ is defined by $y_{i}=y_{j}\to p_{0}\left(j|i\right)=1$ and $y_{i}\neq y_{j}\to p_{0}\left(j|i\right)=0$, the conditional distribution over the inputs $\hat{p}$ is defined as $e^{-d_{ij}}/\sum_{q\neq i}e^{-d_{iq}}$ in which i≠j and $d_{ij}={\left(x_{i}-x_{j}\right)}^{T}\Sigma \left(x_{i}-x_{j}\right)$ is the Mahalanobis distance, where Σ is the data covariance matrix.

**Deep learning features:** in this method, a multi-layered neural network is used to learn high-level features from the different hierarchical transformations of the data through the nonlinear processing units, of which each layer is made. These features are optimized via the back-propagation error minimization procedure. *Autoencoders* can be used to learn a compressed but representative encoding (representation) of the input data. This is achieved by learning an approximating identity function so that the output of the network is similar to the training input. This can be achieved by programming the network to minimize a reconstruction error (e.g., a squared input-output error). The resulting set of reduced features can serve as input for any other machine learning classifier. One disadvantage of auto-encoders with respect to metric learning is that they are not designed to integrate supervised information during learning. A solution is to enable a *Discriminative Autoencoders* (DAUT) by adding the standard cost function of the reconstruction error, the error of the classification can be defined as:

(5) $$\underset{W_{1},W_{2},H}{min}∥X-W_{1}\;H\;X{∥}_{2}^{2}+\lambda ∥Y-W_{2}\;H\;X{∥}_{2}^{2},$$

and solve this equation, where $W_{1}$ and $W_{2}$ are the reconstruction and classification weights, and *H* an encoding matrix and λ a parameter that weights the contribution of each error. The cost function in Equation (5) can be solved by any standard optimizer. Mapped samples can be obtained by the dot product of the original input samples by the encoding matrix. The optimal value of λ can be computed analytically by the l-curve method [31] .

### 3.4. Transitional Activity Tag Classification

For tag classification, in the present work, the machine learning methods presented in the following sections are employed to analyse their performance on recognizing the true label of the activity tag from RA patients with a single accelerometer sensor.

#### 3.4.1. Proposed Novel Meta-Learning Method: *Dichotomous Mapped Forest (DMF)*

In this section, we propose a novel ensemble meta-learning algorithm denoted as Dichotomous Mapped Forest (DMF). The basis of the algorithm provides a solution to the fact that the number of activity tags can be unbalanced as static activities may last for long periods while transitions occur over periods of a few seconds only. Additionally, the heterogeneity between the different activities across subjects and the limits on the available amount of training data challenges the approach of using just a single multiclass classifier. In any case, only eight classifiers are used to build the dichotomy, which is a much smaller number than the necessary for a (one-vs.-one or one-vs.-rest) multiclass schema of 10 classes.

**Figure 3:**
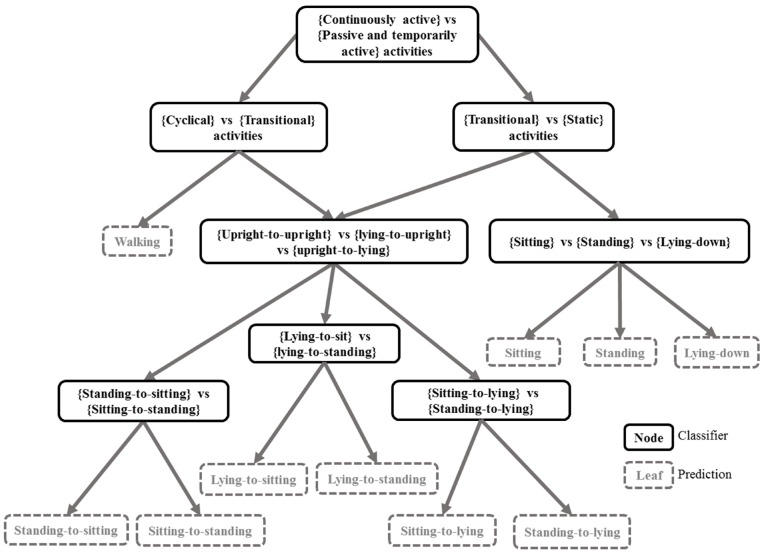
Dichotomy of classifiers in a tree-based structure. Each node corresponds to an independent classifier and each leaf is a prediction. This structure is used to generate predictions as will lately work as a logic tree during the inference process. This will subsequently serve the predictions from all nodes in the dichotomy. In the first splitting node, walking is considered a continuously active activity while sitting, standing and lying-down are categorized as passive or temporally active, while transitions can be initially regarded as both. For transitions, these can be performed in a different fashion, they can be either troubled or comforted, depending on the patient conditions. An untroubled transition should be just a temporarily active activity, but if the patient is afflicted enough this could be slower and unorthodox, resembling a continuous activity. Ergo, transitions require a further splitting from cyclical (walking) on one branch and from static (sitting, standing and lying-down) on the other branch.

Specifically, a dichotomous classifier consists of a hierarchical learning architecture, where in every node an independent classifier is trained with tagged activities from two activity subgroups, with each activity subgroup being represented by a different class to the classifier. The structure of the dichotomy for the activity tags is depicted in Figure 3. It is not necessary to build an ensemble of different dichotomies as we can assess the relationships between the tags by their nature: e.g., continuous passive activities (sitting, standing, lie-down), sudden actions or transitions (sitting-to-standing, lying-to-sitting...) and continuous harmonic activities (walking). Then, going down in the hierarchy, we can narrow down the granularity of nodes into lying-to-upright (lying-to-standing, lying-to-sitting), upright-to-upright (sit-to-stand, stand-to-sit). We employ a random forest (RF) classifier due to its low number of critical hyper-parameters and other advantages such as less sensitivity to outliers and over-fitting. In addition, RF does require a relatively small number of hyperparameters. During training, randomization via bagging and limitation of the maximum tree depth are employed to restrict the complexity of the learned model, thus preventing over-fitting.

A top-down logic hierarchy forming a top-down tree was performed to generate a structure of classification nodes with narrowing specialization. This logic structure is displayed in Figure 3. The subset of data input for each classifier is first mapped into a new data space with higher separability between the classes. This is the same technique used for mapped forests with the difference that we use more complex forms of mapping as described in Section 3.3 rather than principal component analysis.

**Figure 4:**
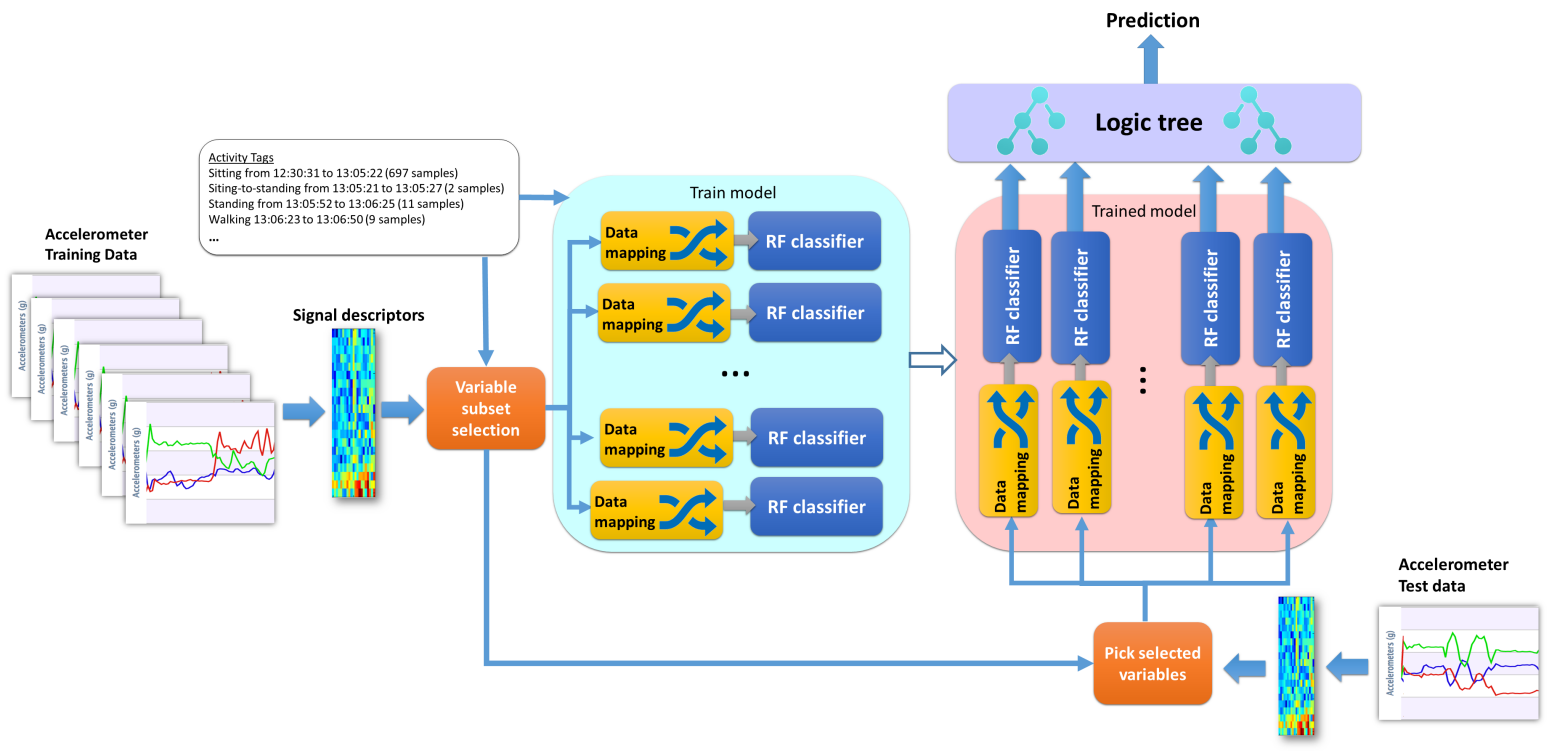
Training and testing architecture of the dichotomous mapped forest. The logic tree at the end of the testing phase is the same as depicted in Figure 3.

The training complexity for this algorithm is slightly higher than a standard random forest but still linear with respect to the number of samples. In big-o notation, this can be $O(\sum_{v}^{g}R\left(m\;n_{v}\;log\;n_{v}\right))$, where *g* is the number of nodes, *R* is the number of random trees and $n_{v}$ the subset of input samples considered in that node. The processing for each node can be easily parallelized in a standard multicore computer by setting a processing thread for each classifier during training and testing. The processing pipeline of the dichotomy is explained in Figure 4 for training and testing. The part indicated as “logic tree” in Figure 4 corresponds to the structure in Figure 3, which has the purpose of integrating the classifier ensemble according to the established hierarchy of the dichotomy. This integration is necessary as each classifier forming part of the dichotomy is indeed a specialized classifier, which can be either binary or multiclass depending on the structure of the dichotomy, but no more than three classes are ever used in a single node.

Each classifier contained in the dichotomy yields an output and confidence relative to the binary classification problem, which is defined by each node of the dichotomy. The confidence output of each classifier in the dichotomy must be fused (Figure 3) in order to obtain a single one for each prediction. Objectively, a suggested way to merge the confidences of the classifiers in the dichotomy is simply to multiply their values as:

(6) $$p\left(y=c|x\right)=\prod_{k=1}^{l}\sum_{u=1}^{g_{k}}I\left(c\in C_{ku}\right)\;p\left(c\in C_{ku}|x,c\in C_{k}\right)\;\;,$$

where *y* is the predicted label, *c* is a specific discrete label, *C* is a subset of labels on a node, where *l* is the number of levels in the *hierarchy*, and $g_{k}$ is the number of nodes on that level. The indicator function I(.) is a Kronecker delta function that returns 0 when $c\in C_{k}$, and 1 when $c\notin C_{k}$. Here, $p(c\in C_{k})$ is bounded between 0 and 1. A limitation of this method is that the confidences of the classifier are multiplying, considering that each node in the dichotomy is indeed independent but not all nodes have the same relevance. That is to say, miss-classifying a sample higher in the hierarchy could lead to more conflicting predictions as many leaves still lie further down. Therefore, higher nodes should play a higher role in the confidence, as the further down we get into the tree dichotomy structure, the closer we are to a leaf. For this, we have developed a new method to weight the confidence that is based on the following equation:

(7) $$p\left(y=c|x\right)=\prod_{k=1}^{l}\sum_{u=1}^{g_{k}}I\left(c\in C_{ku}\right)\;\phi_{k}\;p\left(c\in C_{ku}|x,c\in C_{k}\right)\;\;,$$

(8) $$\phi_{k}=\frac{2\;(l-k+1)}{l^{2}+l}.$$

As a difference with Equation (6), in Equation (7), a weight denoted as ϕ is introduced. These weights become smaller from the upper level node k=1 to the lower level node k=l in the hierarchy and are computed by decreasing function (8).

#### 3.4.2. Machine Learning Classifiers Used for Comparison

Several popular algorithms used in activity recognition with accelerometers are employed in this work for evaluation purposes. In this section, we provide a description about their learning mechanisms as well as advantages and disadvantages.

*Radial Basis Support Vector Machines (RB-SVM).* RB-SVMs are popular for training a classifier and work relatively well with small sets containing few outliers [32]. As its learning mechanism is based on a convex optimization problem, it is resilient to local optima. Its training complexity, $O(n^{2}m)$, is relatively affordable for standard computers, and the number of parameters to predefined are the regularization coefficient and the kernel parameters. The regularization parameter in the SVM controls the complexity of the margin, hence making the model resistant to over-fitting. The bandwidth and regularization parameters are tuned from the training data via cross-validation and Bayesian optimization as provided in several toolboxes [33,34]. Although multiclass versions of SVMs have been developed, better performance has been achieved when binary SVM classifiers are stacked in one-vs.-one or one-vs.-all fashions. However, this latter methodology requires to define appropriate methods for the fusion of the inference from these classifiers. Another limitation of RB-SVMs is that the selection of the regularization and kernel parameters can highly impact the performance of the classifier and its kernel-based models can be quite sensitive to over-fitting [35]. SVMs are essentially a binary classifier in theory. Multiclass SVMs have been claimed not as efficient as their “one-vs.-all” or “one-vs.-one” multiclass schemes [36]. For this application, we need a minimum of 45 SVM binary classifiers.

*Random Forests (RF)* are ensemble algorithms of decision trees that can be applied to any multiclass problem with its standard design. Their learning is based on the concepts of information gain and measuring statistical dispersion rather than kernel-learning as in SVM. They can be applied to many problems given that their most critical parameter for performance is the number of trees, whose value can be easily determined by greedy search or calculating out-of-bag error rates [37]. When the latter is large, it then is necessary to provide the model with more data than with other methods such as SVMs. In terms of their computational complexity, however, it is higher than the one of SVMs but still tractable for standard computers with a few CPU cores, as follows: O(R(mnlogn)), where *R* is the number of trees. They are able to perform implicit feature selection during learning. Although they are not resilient to over-fitting, adding more trees to the forest actually decreases this issue [38]. Over-fitting is controlled by bagging, restricting max three-depth, and the number of potential splits. The effects of applying bagging makes the algorithm more robust with respect to outliers [39]. When working with unbalanced datasets, while SVMs can be tuned to work with penalty weights to penalize the classification of the majority class, and a solution in RFs is a pass for careful management of the data sample partition for each random tree.

*Convolutional Deep Belief Networks (CDBN)* are a neural network type of classifier consisting of stacked unsupervised networks based on restricted Boltzmann machines (RBM) with a Softmax classifier as the output layer. RBM are generative machines whose main finality is to build a generative model of its inputs. It is composed of two layers, namely the visible layer where the input is first introduced and the hidden layer. Each RBM performs several forwards and backwards passes between the visible and hidden layers so as to adjust the connection weights between the input, hidden and output layers in order to be able to generate an output that is very close to the original input data. The difference between the reconstruction and original input is measured by means of KL divergence, which is typically minimized by applying the contrastive divergence algorithms. When RBMs are stacked together to form a DBN, the hidden layer of one RBM is connected to the visible layer of the one above. As a difference from convolutional networks, DBNs present the advantage that they can yield a reasonable performance with just a small amount of labeled data and when a spatial linkage between the input feature is not existent (for instance, a single row feature vector). The partition of the learning into separate RBMs makes the learning process robust against the vanishing gradient issue. In the present work, for CDBNs, the energy function of the RBM has been modified to add a convolutional operator, which ensures the weights are shared among groups of hidden units [40]. This permits standard DBNs to scale to large dimensional inputs, including spatial information, as well as becoming invariant to input transformations by employing a pooling operator. The risk of over-fitting is solved by forcing activation of the hidden units to become sparse, therefore eluding an over-complete model [41]. The Convolutional Neural Networks (CNNs) architecture of learning is optimized to learn from 2D representations of the data. Although signal data from an accelerometer is one-dimensional, some works have proposed using a time frequency representation as input [42].  This is actually performed by computing a *spectrogram* applying a short-time Fourier transform over the three accelerometer forces. This spectrogram represents the power-spectral density component and some studies have reported that inherent information about the movement is encoded in this representation [42]. This transformation of the data aims at converting one-dimensional signal data into a two-dimensional data based on its time-frequency representation. This is actually performed by computing a *spectrogram* applying a short-time Fourier transform over the three accelerometer forces. This spectrogram represents the power-spectral density component and some studies have reported that inherent information about the movement is encoded in this representation [42]. On the one hand, a limitation of this feature with respect to the previous two is that it does not contain class information able to maximize the separability—it is simply a time-frequency representation of the signal. On the other hand, they enable using the data with deep learning architectures such as CNNs.

The disadvantages are that the learning process requires an iterative processing and samplers that may involve a large series of operations; hence, it is recommended to transfer operations such as multiplication between weights and visible or hidden units to a graphical processing units (GPU) or specialized co-processors. Additionally, another disadvantage of these models is that they require pre-defining a large number of hyper-parameters: number of hidden units, weight decay, weight initialization, activation sparsity, learning rate, length of the filter, length of feature maps for both the visual and hidden layers, and so on. Thus, performance is highly dependent on computationally expensive multi-objective optimizers, which may add a significant amount of processing time and machine resources. As standard, hyper-parameters are set to the values suggested in previous works [40].

*Continuous Hidden Markov Models (HMMs)* are effective models that have long been used in the field of physical activity recognition [43,44]. They are machine learning algorithms for sequential data, particularly sequences of discrete samples. The idea behind HMMs is to learn the likelihood of a sequence of observations given a sequence of hidden states. To this aim, an emission and a transition probabilities between a set of hidden states structured in a Markov chain model are learned from the labeled data using maximum likelihood. To estimate the probability that a sequence of observations can be produced by a particular HMM, backwards and forwards inference are performed by means of a dynamic programming algorithm [45]. When working with signals such as G-forces from an accelerometer, it is necessary to first discretize them into a code-book of discrete samples. These can be obtained by quantization or defining a set of clusters (for instance Gaussian mixtures) in the data and an observation as the label of the cluster to which a sample belongs. A metric of optimal cluster dispersion can be used to assess the optimal number of clusters. Clusters’ co-variances are adjusted for positive definiteness and the maximum number of possible hidden states is set to a limit, hence avoiding highly complex models and evading over-fitting. The advantage of the cluster method is that it can handle samples with multiple dimensions. HMMs are not very expensive to train in modern computers and their parametrization is not excessive as their only critical parameter is the number of hidden states, which can be estimated by any simple greedy or heuristic search. However, as HMMs are inherently designed to be trained with discrete inputs, the performance is highly affected by how well the discrete samples can represent the nature of the original continuous signal.

### 3.5. A State Machine as a Logic Filter

**Figure 5:**
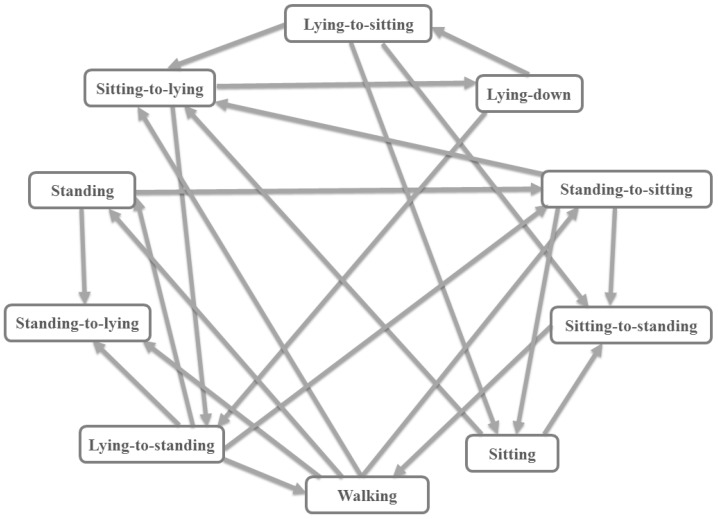
State machine of the logical filter with allowed connections between activity predictions. For instance, once a sample input, representing 3 s, has been predicted as “stand-to-sit”, a “stand”, “lying-to-stand“, “sit-to-stand” or “walking” should have previously been predicted.

Most clinical studies present a set of activity tags of interest rather than a universal set. These activity tags are related since a set of activities can occur sequentially in time in a determined order of the logic filter; for instance, one could expect a sit-to-stand prediction after a sitting position but not a stand-to-lying. The number of previous predictions checked to find one of these allowed activities is regulated by the order of the logic filter. In addition, in the case when this is incorrectly predicted, our hypothesis is that a well calibrated classifier should output a low confidence for this prediction. Then, we can check whether this sequence is possible in our activity state machine every time our classifier outputs a prediction with a confidence level lower than a determined threshold. The state machine of possible activity sequences is presented in Figure 5. Most of the transitions between the states of static activities account for a transitional activity in between them, although some exception is made for walking, for which direct transitioning to a static activity state might be permitted, for instance passing from walking to simply standing. Most classifiers yield a confidence value, sometimes in the form of firing strength, probability or distance to a separating plane. In the case of the DMF, this can be done by merging the confidence of all classifiers that form part of the dichotomy, using Equation (7). Figuring out a lower bound, we denote a δ, on the confidence level can be performed by leaving aside a small set of labeled data from the training set, obtaining their predicted values and confidences and finding the optimal operating point among the points in a receiver operating characteristic (ROC) curve. An automatic criterion is to pick out the point corresponding to the minimum Euclidean distance between the point (0,1) and the points in the ROC,

(9) $$\delta =min\left(dist\left(i\right)\right)\;;\;dist\left(i\right)=\sqrt{ROC_{x}^{2}+{\left(1-ROC_{y}\right)}^{2}}\;\;,$$

where $ROC_{x}$ and $ROC_{y}$ are the *x* and *y* values of the ROC curve.

The threshold on the confidence sets a trigger to activate the logical filter of the predictions by looking at the activity automata (Figure 5). This can be effectively achieved by checking the constraints between impossible transitions. If the predicted confidence bound is not surpassed, then the following condition is evaluated:

(10) $$\beta :=\left(S_{t-1}\neq \;S_{t}\right)\;\;\wedge \;\;p\left(y_{t-1}=c|x\right)<\delta \;\;\wedge \;\sum_{i}^{order}\left(S_{t-(i+1)}\cdot \Theta \cdot S_{t-1}\right)\;\;\;\wedge \;\;\left(S_{t-1}\cdot \Theta \cdot S_{t}\right),$$

(11) $$\left\{\begin{array}{cc} \beta =true & y_{t-1}:=y_{t-1},\\ \beta =false & y_{t-1}:=y_{t-2}, \end{array}\right.$$

where $S_{t}$ is the state of the current time. The state is a single-entry vector where all the elements are zero apart from one which corresponds to the class index of $y_{t}$. The symbol Θ is an adjacency matrix of logical true or false values, indicating whether the transition conditions of the finite state-machine are possible. The factor order allows for a level of flexibility in searching for the valid transition between the previous transitions. If a valid sequence is found between the previous activities until $t-\left(order+1\right),$ then the same label is kept despite not surpassing the lower bound on the confidence.

Algorithms 1 and 2 show the sequence of processing steps for training and testing the DMF. Symbols *X* and *Y* are the input and tag label data, respectively, and $Sel_{node}^{feat}$ are the selected features for each node in the dichotomy after applying SFFS. $C_{node}$ is the list of confidence of the prediction for each node and $C_{node}^{norm}$ represents the ones normalized through the dichotomy.
**Algorithm 1** Dichotomy mapped forest—Training**Input:**  *X*,*Y* **Output:**  trained model, map & $Sel_{node}^{feat}$ 1:**for** Each node in logic three according to the dichotomy in Figure 3 **do**2:    Split *X* into $X_{A_{node}}$ and $X_{B_{node}}$ acording to the node label grouping in each node in Figure 33:    [$X_{A_{node}}^{sel}$,$X_{B_{node}}^{sel}$,$Sel_{node}^{feat}$] ← call SFFS (input=[$X_{A_{node}}$,$X_{B_{node}}$], labels = [$A_{node}$,$B_{node}$])4:    **if** mapping == DL **then**5:        [map(node),$mapped_{A}$,$mapped_{B}$] ← call DAUT (input = [$X_{A_{node}}^{sel}$,$X_{B_{node}}^{sel}$], labels = [$A_{node}$,$B_{node}$])6:    **else if** mapping == Metric **then**7:        [map(node),$mapped_{A}$,$mapped_{B}$] ← call MCM (input = [$X_{A_{node}}^{sel}$,$X_{B_{node}}^{sel}$], labels = [$A_{node}$,$B_{node}$])8:    **end if**9:    model(node) ← call train RF (input = [$mapped_{A}$,$mapped_{B}$], labels = [$A_{node}$,$B_{node}$])10:
**end for**


**Algorithm 2** Dichotomy mapped forest—Test**Input:**  *Z* (test set), model, $Sel_{nodes}^{feat}$, map **Output:**  $Y^{pre}$
1:**for** Each node in logic three according to the dichotomy in Figure 3 **do**2:    $Z_{node}^{sel}$ ← select $Sel_{node}^{feat}$ features in $Z_{node}$3:    ${mapped}_{Z}$ ← map input $Z_{node}^{sel}$ with map(node)4:    [$Y_{node}^{pre}$,$C_{nodes}$] ← test ${mapped}_{Z}$ in model(node)5:
**end for**
6:[$Y^{pre}$,$C_{nodes}^{norm}$] ← apply Dichotomy (predictions = $Y_{nodes}^{pre}$, confidence = $C_{nodes}$) as Figure 3 & Equation (7)7:${bound}_{nodes}$ ← get confidence bound using $C_{nodes}^{norm}$ as Equation (9)8:**if** $C_{node}^{norm}$ < ${bound}_{node}$ **then**9:    $Y^{pre}$ ← check logic filter as in (11)10:
**end if**



### 3.6. Training Settings: Mixing Patients and Healthy Volunteers

**Figure 6:**
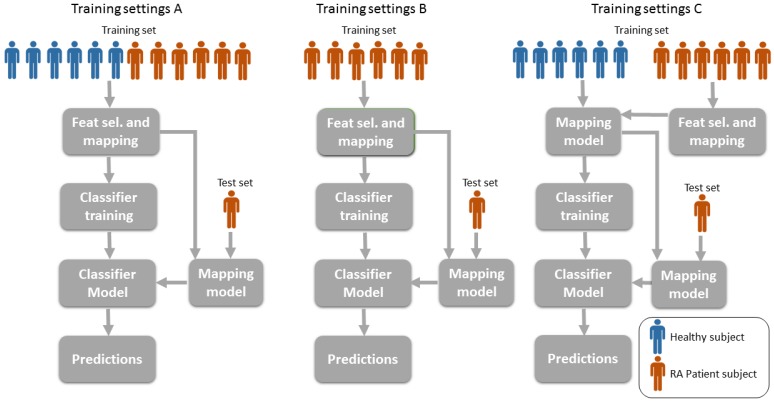
Training settings of the classifier. In setting A, data from healthy volunteers is appended to the patient dataset to increase its size. In setting B, only patient data is used to train the classifier model. Finally, in setting C, data from the patient population is used to find the most relevant features and build the mapping; then, the model is trained with healthy data only. In all cases, only patient data is employed to generate the predictions. As one would expect, the data used for tests will come from a different set than the one used for training.

Clinical studies frequently account for a number of patients and control subjects or volunteers. Patient data concerns a very restricted population and, consequently, a common question when working with patient data is to know whether the dataset can be extended with data from healthy volunteers. Figure 2b highlights the difference in signal patterns between patients and healthy volunteers. In this regard, we aim at investigating the hypothesis that the classification performance of the algorithm can be affected by implementing the training in different ways. Figure 6 shows a graphical representation of the three different training settings.

## 4. Results and Discussion

This section examines the performance of the methods described in Section 3. The machine learning algorithms proposed in Section 3.3 are trained using a *dataset* formed of healthy subjects and patients performing the same activities. The results shown in Section 4.1 and Section 4.2 apply the training setting A as depicted in Figure 6. Inertial data is pre-processed to match the desired input method used for the particular classification method. For instance, RF-based methods can be trained with dimensionality reduced inputs from either deep learning (DL) or metric learning (Metric), and the same is applicable to the SVM-based classifiers. CDBN methods are trained using a 2D spectrogram representation of each signal axis as suggested in [46]. For C-HMM, the input features are first quantized to form a transcript (codebook) proposed methods described in [47].

### 4.1. Activity Classifiers Comparison

**Figure 7:**
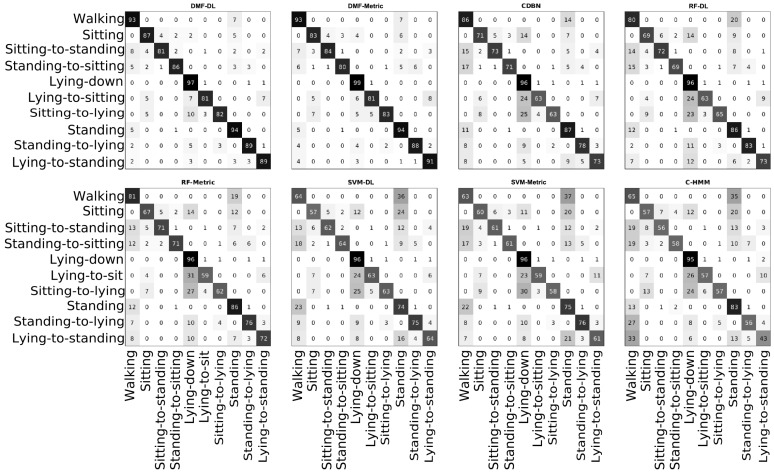
Confusion matrices showing predictions as rows and ground truth labels as columns for eight different methods. Numbers inside the confusion matrices represent percentages over the total number of samples predicted as a specific tag.

This section provides the results of classifying activity patient data using leave-one-subject-out cross-validation to test for the subject independence of the learned model. Figure 7 shows the confusion matrices for each method, which are ordered by decreasing performance from left to right columns and from up to down rows. A major number of segments classified with true activity tags are found in the main diagonal for the suggested best method DMF. Accuracy, recall and specificity are above 90% for the DMF-based methods (Table 1), using inputs spanned by a subspace mapping generated by either a DAUT or metric leaning. However, the F-scores are rather moderate, namely approximately 74% and 73% for DMF-DL and DMF-Metric, respectively. Furthermore, activities that involve transferring from a lying to a stand position are amongst the hardest for the RA patients because they entail a range of sub-movements including pelvis and lumbar torsion as well as joint exertion, which are triggers for pain. The level of persistent muscular pain and stiffness caused by the disease has an important impact on the way patients perform these activities. Additionally, the tagging of the latter is complex as the patient may perform the movements task as a sequence of smooth movement to reduce the feelings of muscular pain, so that rather than just being a single lying-to-stand, it can be seen as a composite of subtle supportive actions until the patient finally achieves a comfortable standing position. Given that the accelerometer cannot provide a measure of angular displacement, if the patient performs repeated balancing and little jumps when transitioning towards the upright position, the signal might exhibit some repeated burst of acceleration and predictions could be likely inaccurate. Likewise, if the patient is performing long gaps of static motion while transitioning, this could be predicted as a static activity.

Table 1 shows the numerical results from the performance test, the best two approaches being DMF-DL and DMF-Metric. Upon statistical test using non-parametric Kruskall–Wallis and Dunn’s test multiple comparison, the performance of DMF-DL is significantly different from SVM-DL and any other classifier down-performing it with (*z* = 49.80, *p* < 0.01).Likewise, DMF-Metric is significantly different from SVM-Metric and any other classifier down-performing it with (*z* = 81.18, *p* < 0.01). Alternative statistical tests using analysis of variance and Tukey-kramer test show that DMF-DL and DMF-Metric are statistically different from CDBN and any other classifier down-performing it with (*q* = 0.19, *p* < 0.01) and (*q* = 0.19, *p* < 0.01), respectively. The performance of DMF-Metric and DMF-DL are not significantly different.

### 4.2. Applying the Logic Filter

A requirement for an actigraphy is that the sequence of activities must be logical for the activity tags defined. The method described in Section 3.5 is applied for the best two methods from Section 4.1 (DMF-DL and DMF-metric). DMF-DL and DMF-metric are the only methods that exhibit both high accuracy and a moderately good F-score as shown in Figure 7 and Table 1. This means that, although most of the ground truth tags are correctly predicted as true positives and true negatives, several other predictions are false positives. As discussed in Section 4.1, this could be produced by the imprecision of the tags; however, a way of ensuring that our tags follow a valid sequence is to activate the logic filter for the prediction as presented in Section 3.5.

**Figure 8:**
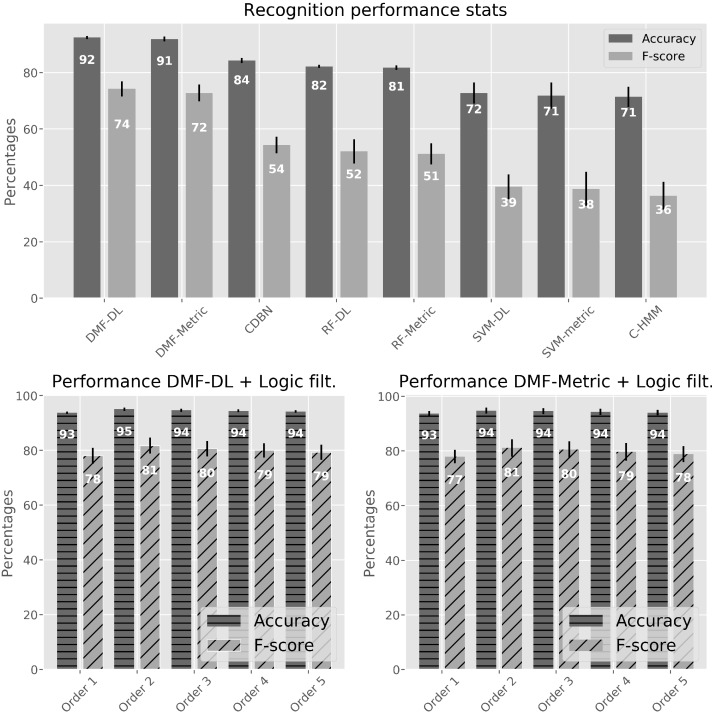
Performance statistics. Upper part: performance results (accuracy and F-score) for the methods in Table 1; Lower part: performance results for DMF-DL and DMF-Metric applying the logic filter with different orders.

The logic filter considerably improves the values of the F-score for the recognition performance, as it can be seen in the lower part of Figure 8. This is important to obtain a well-balanced classifier. Indeed, the logic filter is able to delete those false positives that do not exhibit a logical sequence. The order providing the best results is two, and this means that checking two previous predicted activity tags is enough to find an allowed sequence. While the following orders also have a good impact on the overall classification, the performance either remains the same or decreases (Table 2). Looking for a coherent sequence in many past tags might indeed result in inconsistent logical assumptions.

**Figure 9:**
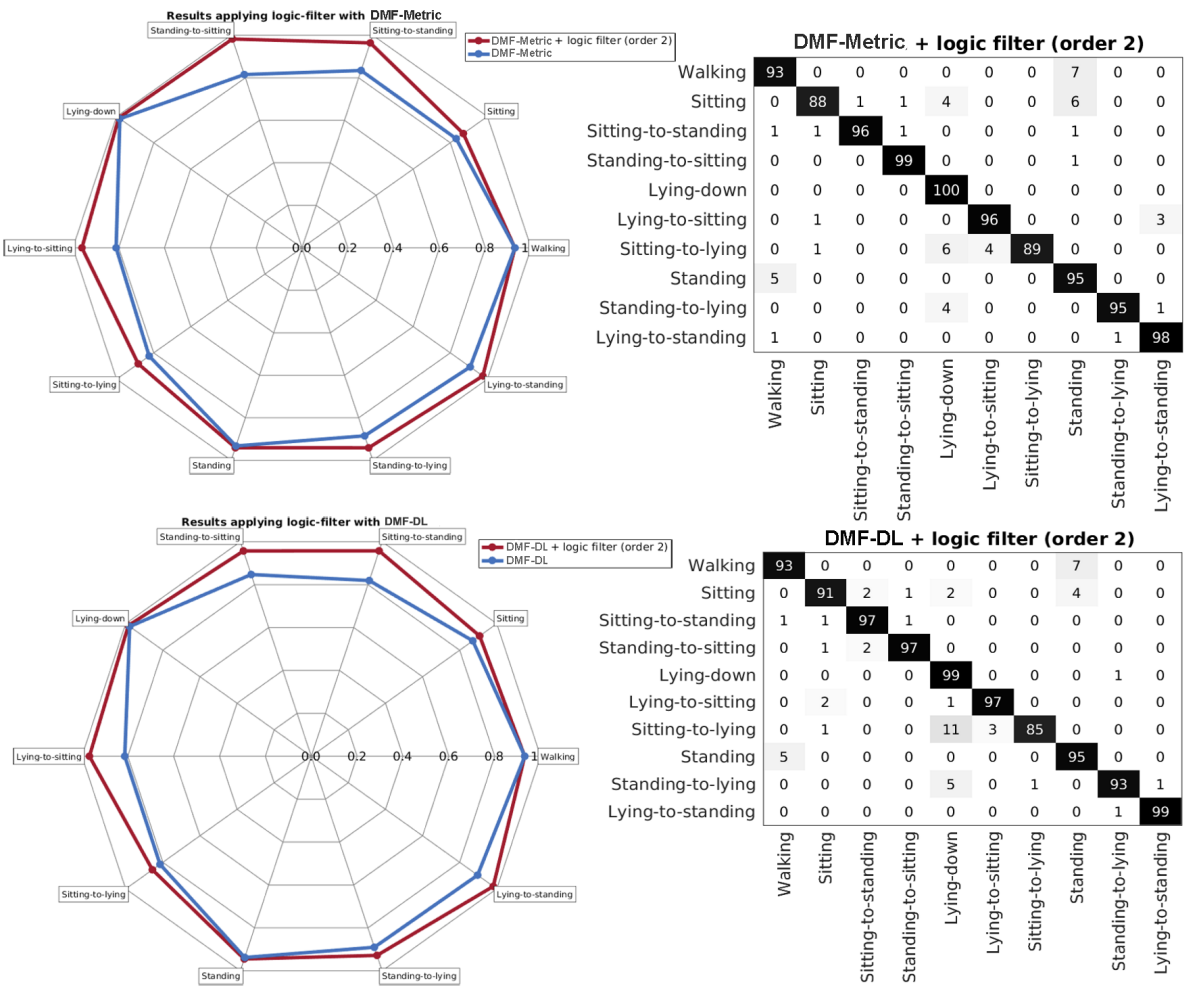
Performance results for applying the logical filter to DMF-Metric (upper) and DMF-DL (lower). The spider diagram shows the percentage of correctly classified input for each independent activity tag.

In Figure 9, a comparison between the performance obtained without applying the logic filter and the one obtained after applying it is shown. As it can be seen, the activity tags that benefit the most are the lying-to-upright transitions as the filter exploits the high sensitivity (above 85%) of recognizing the preceding static activities such as stand, sitting, or lying-down. Overall recognition rates for other transitions such as sit-to-stand and stand-to-sit also improve after applying this correction. Recognition rates are high (over 80%) for all considered activity tags. The differences between applying the mapping powered by the DAUT (DL) or MCM (Metric) are not significant.

### 4.3. Results with Different Training Settings

In this section, the best two classifiers, i.e., DMF-DL and DMF-Metric, are trained with different subsets of the data. In particular, we use the settings described in Section 3.6. Previous results from Section 4.1 and Section 4.2 were performed using training settings A as described in Figure 6, which consists in using all the available data to train the classifiers. Patient data is difficult to collect as clinical trials require a larger amount of work in terms of preparation as well as the need for an assisting medical team during the study. Hence, one might be interested in looking at the capabilities of the classifier with different training approaches such as using just patient data for training or just for the feature mapping.

Table 3 shows how the training is performed applying settings B and C from Figure 6. It can be seen that, using the patient data only, the performance of the DMF-DL method is diminished by 17% in accuracy and 40% in F-score while the performance of DMF-Metric decreases by 13% in accuracy and 31% in F-score. When applying the patient data just for the mapping, the performance results also lowers the classifier performance, although less than when using patient data only for training. This deterioration in the results reaches 12% in accuracy and 30% in F-score for DMF-DL, compared to 9% and 25% for DMF-Metric. If the logic filter is applied for the predictions, then the negative impact on the results is less dramatic. Specifically, using just patient data for training worsens the results by 8% in accuracy and 26% in F-score for DMF-DL, while the performance decreases by 5% and 16% for DMF-Metric. When the patient data is used for mapping only, then the lowest deterioration is achieved with 5% decrease in accuracy, but 16% in F-score for DMF-DL, while, for DMF-Metric, the deterioration is just 2% in accuracy, but 15% in F-score.

The conclusion from this test is that within the limits investigated, the more (either patient or healthy) subject data is used to jointly train the mapping and the classifier, the more accurate the predictions are. Alternative settings such as B and C are not recommended because they penalize the classification performance for the best approach, particularly the F-score estimates. DMF-Metric exhibits a slightly higher average performance than DMF-DL for this test, and the performance differences between DMF-DL and DMF-Metric are not significant either.

## 5. Conclusions

In this work, data from a single accelerometer was used to classify static, acyclic and transitional activities. Several methods were adopted as a comparison to analyse the performance behaviour from different machine learning classifiers. Using discriminatory mapping as the ones described in Section 3.3, i.e., which uses the labels from the training data to obtain an optimal subspace to discriminate between activities, proved to be a solution to cope with the signal variations between subjects, particularly the ones arising from the congruity of the inertial data from healthy individuals and the diversity among the RA patients. Building a single multi-classifier from a highly diverse sequence of activities that can also have particular divergences across subjects is a challenging task. We have found positive the utility of an ensemble of classifiers as a form of dichotomy of nodes consisting of a single classification task and described in detail. The division of activities follows a quasi-logical structure, permitting most general conceptual division of activities such as active versus passive activities to be discriminated in advance of specific ones such as sitting or stand-to-sit and the like. It is worth noting that standard ensemble learning depends on the randomization of the input data, which, in this case, may not be as advantageous, as an arbitrary arraignment would ignore the intrinsic categories among activities.

As another relevant finding, when a substantial number of activity tags (ten in the present study) is requested to be classified, with just one sensor and an uncertain validity of the tags, then adding a prediction corrector in the form of a logic filter on top of the less confident predictions helps to achieve more accurate results, as the desired activity tags are always expected to follow a rational and valid sequence.

The best classifications were achieved with the DMF methods, consisting of the dichotomy and mapping plus the classifier. There were no statistical differences between the performance of both types of mapping, the DL mapping being slightly above the one of MCM when all the data in the study is provided to the classifier. It is worth mentioning that the former requires more computational resources than the later so that a powerful GPU computing, dedicated co-processors or a computer cluster are recommended. In the present study, we experienced that the gain of using DL mapping with respect to MCM mapping is not sufficient to justify the higher computational cost for this specific application, as it can be seen from the results in Section 4.2. The best results were approximately 95% accuracy and 81% F-score. These results are also dependent on the evaluation method used. Leave-one-subject-out cross-validation was the chosen method in order to avoid randomly partitioned k-folds, which may end-up including singular activities from the same subject in both the training and test set, hence jeopardizing the subject independence of the learned model.

Although we have intended to simulate a very realistic environment during the experiment, including different types of chairs and walking speeds, to allow natural motion of the subject etc., we might expect that during free-living there could be a set of different scenarios that we cannot replicate during our lab experiments. Nevertheless, the proposed method is designed with robustness in mind to avoid nonsensical predictions by means of the logic filter as well as avoiding signal instabilities by virtue of the proposed pre-processing and subsequent data mappings. Therefore, we do have the certainty that although the accuracy of “free-living” activity predictions might not be identical to the ones reported in this study, they will not degrade significantly. The set of activities provided during training will be recognized to the same extent. The variability of the position of the sensor can be an additional limiting factor during free-living. In previous clinical experiments involving inertial sensors, the lower back position at the L3 to L5 vertebrae has been a popular location due to its closeness to the center of mass [48,49], and acceptable for long-term studies at-home use [50,51]. Nevertheless, proper fitting of the sensor by clinical professionals during the first visit would minimize the impact of possible sensor detachment or displacement.

To conclude, this study showed that it is possible to recognize activities from individuals with disabilities affecting their mobility and postural condition such as RA, in short periods of three seconds, from a single accelerometer attached to the L5 with compelling accuracy. The methods presented in this work can be helpful for the elaboration of clinical studies where wearable data can be used to build actigraphies from patient data.

Last but not least, we would like to highlight for future research the potential of sensors providing novel quantitative physiological bio-markers in order to monitor the health status of the musculoskeletal system of the patients. Along these lines, a novel and promising wearable sensor with contact microphones to pickup acoustical emission from joint sounds has recently been proposed [52], and there are also advances for the in-vivo monitoring of stresses and strains of musculoskeletal tissues by implantable sensor technologies [53,54]. These new sources of local health status in combination with accurate daily-living actigraphies may potentially yield a rich source of information enabling a personalised disease management and optimal care.

## Figures and Tables

**Table 1:** Performance values for the methods in Figure 7.

Statistics	DMF-DL	DMF-Metric	CDBN	RF-DL	RF-Metric	SVM-DL	SVM-Metric	C-HMM
Accuracy	92.44 ± 0.54	91.95 ± 0.82	84.30 ± 0.98	82.25 ± 0.55	81.76 ± 0.86	72.75 ± 3.73	71.84 ± 4.67	71.40 ± 3.6
Sensitivity	92.20 ± 1.98	90.82 ± 1.98	78.88 ± 1.14	81.42 ± 1.66	80.87 ± 0.62	74.37 ± 2.06	73.78 ± 0.88	68.12 ± 2.61
F-score	74.22 ± 2.63	72.76 ± 2.96	54.33 ± 2.94	52.02 ± 4.29	51.21 ± 3.75	39.48 ± 4.42	38.70 ± 6.10	36.32 ± 4.94
Specificity	92.46 ± 0.55	92.13 ± 0.96	85.04 ± 1.05	82.35 ± 0.67	81.88 ± 0.97	72.49 ± 4.15	71.64 ± 5.31	71.85 ± 4.92

**Table 2:** Statistical performance results for DMF-DL and DMF-Metric with activated logic filter.

Algorithm	Statistics	Order 1	Order 2	Order 3	Order 4	Order 5
DMF-DL	Accuracy	93.78 ± 0.45	95.00 ± 0.65	94.64 ± 0.55	94.43 ± 0.50	94.21 ± 0.43
	F-score	78.05 ± 2.93	81.77 ± 2.90	80.63 ± 2.82	79.97 ± 2.67	79.30 ± 2.65
DMF-Metric	Accuracy	93.75 ± 0.88	94.76 ± 1.15	94.56 ± 1.10	94.31 ± 1.11	94.06 ± 1.01
	F-score	77.92 ± 2.50	81.12 ± 3.20	80.44 ± 3.13	79.67 ± 3.23	78.87 ± 2.97

**Table 3:** Classification results with settings B and C from Figure 6.

Algorithm	Without Logic Filter		With Logic Filter	
	Accuracy	F-score	Accuracy	F-score
DMF-DL patient data only (setting B)	78.01 ± 0.96	43.78 ± 3.72	84.11 ± 1.12	53.69 ± 3.22
DMF-DL patient mapping (setting C)	83.12 ± 1.42	52.31 ± 3.7	88.84 ± 4.55	63.88 ± 4.55
DMF-Metric patient data only (setting B)	81.45 ± 1.28	50.39 ± 3.86	87.63 ± 1.65	60.98 ± 4.63
DMF-Metric patient mapping (setting C)	85.06 ± 0.95	55.75 ± 4.16	90.37 ± 1.15	61.79 ± 4.46

## Abbreviations

The following abbreviations are used in this manuscript:

C-HMM	Continuous Hidden Markov Models
CDBN	Convolutional Deep Belief Networks
DCNN	Deep Convolutional Neural Networks
DL	Deep Learning
DMF	Dichotomous Mapped Forest
DMF-DL	DMF with Deep Learning mapping
DMF-Metric	DMF with Metric learning mapping
DAUT	Discriminatory Autoencoder
GPU	Graphical Processing Unit
HV	Healthy Volunteers
KL	Kullback-Leibler
L5	L5 lumbar vertebra
MCM	Maximally collapsing metric
MEMS	Microelectromechanical systems
Metric	Metric Learning
RBF	Radial Basis Function
RF	Random Forest
ROC	Receiver Operating Characteristic
RBM	Restricted Boltzmann Machine
RA	Rheumatoid Arthristis
RMS	Root Mean Squared
SFFS	Sequential Fowards Floating Selection
SVM	Support Vector Machine
